# Impaired axon regeneration and heightened synaptic dynamics in the injured aged mammalian cortex

**DOI:** 10.1016/j.isci.2025.113665

**Published:** 2025-09-30

**Authors:** Cher Bass, Anil A. Bharath, Vincenzo De Paola

**Affiliations:** 1 Centre for Neurotechnology, Imperial College London, London SW7 2AZ, UK; 2 Department of Bioengineering, Imperial College London, London SW7 2AZ, UK; 3 Institute of Clinical Sciences, Faculty of Medicine, Imperial College London, London W12 0NN, UK; 4 Duke-NUS Medical School, 8 College Road, Singapore 169857, Singapore; 5 Brain Sciences Department, Faculty of Medicine, Imperial College London, London W12 0NN, UK

**Keywords:** Molecular neuroscience, Cellular neuroscience

## Abstract

Aging is a key risk factor for impaired neural repair, yet its effects on axon regeneration and synaptic remodeling in the brain remain unclear. To investigate age-related repair mechanisms in neural circuits, we developed an axonal injury model in the aged (>2 years) mouse somatosensory cortex and tracked fluorescently labeled axons using *in vivo* multiphoton imaging. Axon degeneration rates were similar to young adults, but regeneration was markedly reduced. Six hours post-lesion, *en passant* boutons (EPBs)—the most common cortical synapse—showed transient increases in number and size. To assess functional consequences, we used a recurrent neural network model to simulate memory dynamics, revealing distinct changes compared to young adults. Our results suggest that increased synaptic turnover in the aged brain may facilitate partial recovery from injury through synaptic re-wiring, highlighting potential mechanisms supporting neural adaptation in aging.

## Highlights

• Impaired axon regeneration in the aged brain is not due to slowed degeneration
• Synaptic reorganization occurs following axon injury in the aged brain
• Synaptic reorganization may serve as a compensatory mechanism after injury

## Graphical abstract

**Figure:**
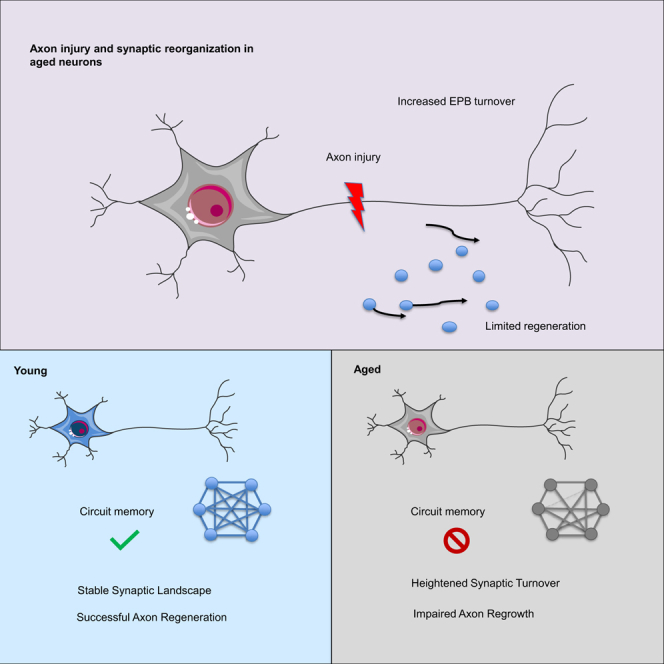

## Introduction

Aging is associated with impaired brain functions, including decline in sensory abilities^1^^,^^2^ and cognitive function,^3^^,^^4^^,^^5^ as well as increased vulnerability to injury or disease.^6^^,^^7^^,^^8^ However, the underlying mechanisms remain poorly understood. Understanding how aging influences the brain’s ability to repair and adapt after injury is critical for developing effective therapeutic strategies to promote recovery and improve the quality of life in an aging population. Aging has an established detrimental effect on nerve recovery after injury in the spinal cord,^6^^,^^9^^,^^10^ but its effect in the injured brain is less clear. For instance, the slow degeneration of the distal portion of the axon following injury, and the clearance of axon fragments have been investigated in the peripheral nervous system (PNS) and spinal cord.^11^^,^^12^^,^^13^^,^^14^^,^^15^^,^^16^ Evidence from PNS suggested that slower debris clearance of degenerated axons leads to impaired regeneration.^13^^,^^16^ Also, regeneration is improved when degeneration (of the distal axon) is induced before or simultaneously with an axon lesion.^14^^,^^15^ Axon degeneration and regeneration in the brain are challenging to study as many current techniques rely on fixed tissue analysis, which provides only a snapshot of degeneration and requires a large number of animals. Real-time, dynamic imaging is needed to understand the sequence of degeneration and repair processes in the brain.^17^^,^^18^

In our previous work, we investigated how injury affects cortical axons in the young adult brain, and found a cell-type specific response where En Passant Bouton (EPB)-rich axons regenerated less (20%) than Terminaux Bouton (TB)-rich axons (55%).^11^^,^^12^ We also compared young adult (2–3 months old) and aged mice (*>* 2 years old) and found an increase in axonal bouton dynamics in a subset of cortical neurons.^19^ EPB-rich axons, which originate in Layer (L) 2/3/5, or the thalamus, rather than TB-rich axons, which originate in L6, increased their turnover rate (TOR; i.e., their formation and elimination), and rate of size change with age.^19^ Thus, aging may impact cognitive impairment^5^ by affecting synaptic re-wiring.^19^^,^^20^

Here, we investigated how axonal injury affects axonal and synaptic responses in the aged somatosensory cortex with longitudinal *in vivo* multiphoton microscopy. We used laser-mediated axotomy, a technique known to cause a small local lesion that induces only transient microglia recruitment and minimal glial scar formation.^11^^,^^12^^,^^21^ While this lesion paradigm does not replicate the complexity of injury processes in diseases such as traumatic brain injury,^22^ it provides a valuable model for studying the cell-intrinsic mechanisms underlying axonal reorganization after injury,^23^ without the need to sacrifice the animals. We focused on EPB-rich axons, known to be affected by aging^19^ to investigate whether their response to injury differs from that of the young adult brain. Using time-lapse imaging from 4 days pre-lesion to 3 months post-lesion, we examined regeneration, degeneration, and synaptic reorganization. Our findings revealed impaired regeneration in the aged brain, along with increased synaptic dynamics after injury. A computational model allowed us to explore whether these dynamics could be compensatory, showing that they may allow for partial recovery in a memory task following injury. This approach provides valuable insight into cellular and synaptic responses to axonal damage during aging and establishes a foundation for investigating potential therapeutic targets in the aged brain.

## Results

**Figure 1:**
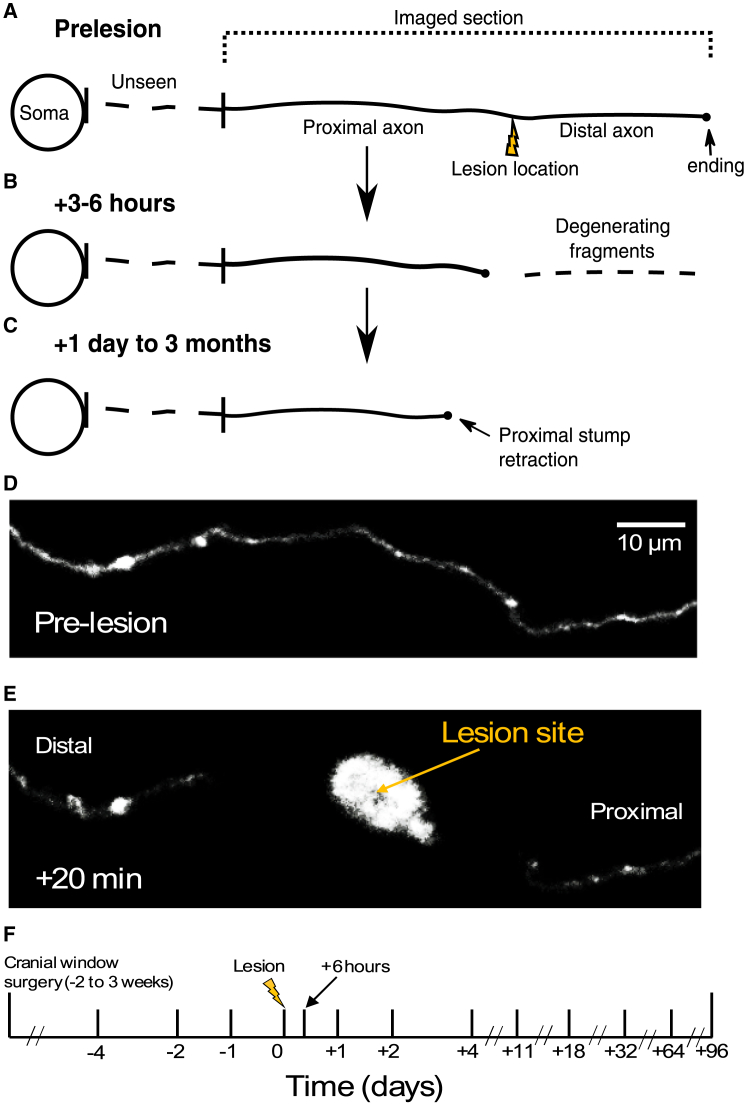
Tracking axonal dynamics following lesion in the aged brain: Degeneration, retraction, and regeneration (A) An axon of suitable length is selected before the lesion. The lesion location is chosen, and the axon is measured. The proximal portion (closer to the soma) is imaged to track retraction and potential regeneration. The distal portion is monitored until degeneration is complete. (B) By 6 h post-lesion, fragmentation of the distal axon is typically observed. (C) Degeneration of the distal axon is generally completed within one day post-lesion. The proximal axon is imaged for up to 3 months to assess retraction, regeneration, and synaptic dynamics. (D) Example of a pre-lesion axon image. (E) A representative response at 20 min post-lesion, showing the comparable axon loss of both proximal and distal portions from the lesion site. (F) Imaging schedule showing time points used to track degeneration, regeneration, and synaptic dynamics up to 3 months post-lesion.

To investigate the responses of cortical axons and their synapses to localized injury, we used a multiphoton imaging femtosecond laser to selectively induce lesions by locally increasing its power.^24^ The typical axonal response to injury and the imaging timeline are depicted in Figure 1. Pre-lesion imaging was used to select axons of appropriate length and type, particularly EPB-rich axons originating from layers 2/3/5 and thalamocortical projections (Figure 1A). On the day of the lesion (day 0), these selected axons were subjected to laser-mediated axotomy (Figures 1B and 1E). Post-lesion, these axons were tracked for up to 3 months to assess their regenerative capacity, degeneration processes, and synaptic dynamics over time (Figures 1C–1F).

We followed EPB-rich axons (*n* = 14, all experiments) over multiple time-points (Figure 1F) over days and months with *in vivo* two-photon imaging. Lesion sites along the axons (*n* = 14) were selected to create disconnected axons with lengths comparable to previous studies (∼150–500 μm, average 314 ± 111 μm). The total visible axon length ranged from ∼400 to 1500 μm (average 738 ± 317 μm). Post-lesion, axons exhibited a typical response, including retraction from the lesion site (Figures 1B–1E; retraction length 18 ± 4 μm, 20 min post-lesion) and an auto-fluorescent mark left by laser damage (Figure 1E; lesion mark diameter 9 ± 2 μm, 5 min post-lesion). Successful axotomy was confirmed by examining the axons at subsequent time points, where the degeneration of the distal axon portion was observed (*n* = 14). In all cases, the proximal portion of the axon remained intact and was subsequently imaged at later time points. This allowed for the analysis of its retraction behavior, potential regeneration, and changes in synaptic dynamics over time.

### Axon regeneration is not observed after injury in the aged cortex

The regeneration of EPB-rich axons was tracked in aged mice for up to 3 months post-lesion. No regeneration was observed from the proximal stump in any of the examined axons (*n* = 0/9). To assess the significance of this regeneration absence, we performed a binomial test based on an expected regeneration rate of 20% (from young mice^11^), which indicated that the probability of observing no regeneration by chance was 0.13 (binomial test), suggesting a trend toward reduced regenerative capacity with age. However, a minor growth was detected in one instance, where a branch (not the proximal stump) from an injured axon extended 16 μm over a period of 3 days.

### Axon retraction rates in the aged brain are comparable to non-regenerating axons in young mice

**Figure 2:**
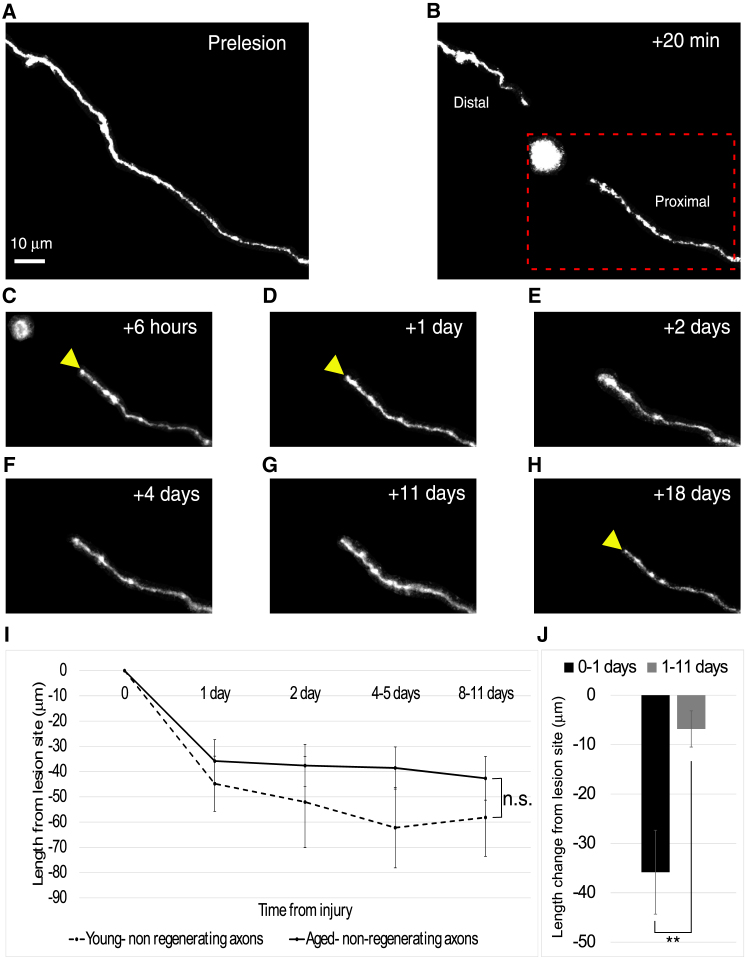
Axonal retraction dynamics in aged vs. young animals following lesion (A) Representative two-photon image of a cortical axon in the aged brain before the lesion. (B–H) Sequential images of the same axon captured at 20 min, 6 h, 1 day, 2 days, 4 days, 11 days, and 18 days post-lesion, respectively. The yellow arrowheads mark the retracted position of the proximal stump. The red dotted box highlights the region of interest consistently followed in panels (C–H). (I) Quantitative comparison of axon retraction distances between aged and young animals, showing no significant difference (*p* = 0.185, Kruskal-Wallis). (J) Retraction occurs in two distinct phases: an acute phase, with significant retraction within the first day post-lesion, followed by a chronic phase with minimal retraction thereafter (*p* = 0.0078, Wilcoxon signed rank). ∗*p* < 0.05; ∗∗*p* < 0.01. Error bars represent SEM.

Axons in the aged brain retracted an average of 53 ± 26 μm over the 3-month observation period post-lesion (*n* = 9; Figure 2). Most of this retraction occurred within the first day (36 ± 25 μm), with slower retraction continuing until 3 months (6.8 ± 11 μm; *p* = 0.0078, Wilcoxon signed-rank). The retraction dynamics in aged mice were comparable to those of non-regenerating axons in young adult mice^11^^,^^12^ (*n* = 53; *p* = 0.185, Kruskal-Wallis). Retraction distances (μm) from the lesion site were as follows.

1. Day 1: Aged: 36 ± 25 vs. Young: 44 ± 79
2. Day 2: Aged: 38 ± 25 vs. Young: 52 ± 93
3. Days 4–5: Aged: 39 ± 25 vs. Young: 62 ± 87
4. Days 8–11: Aged: 43 ± 25 vs. Young: 58 ± 87

**Figure 3:**
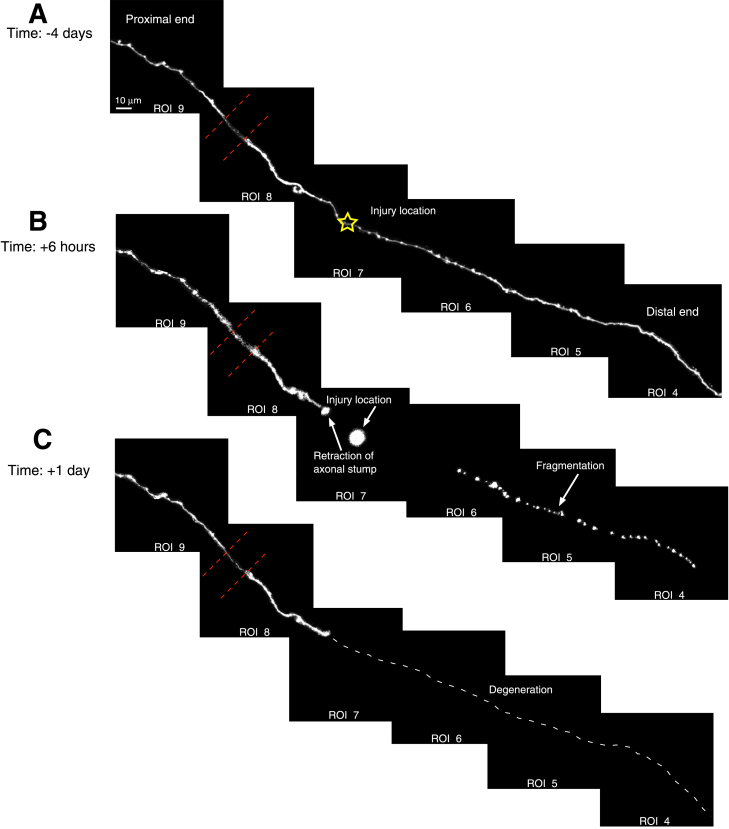
Example of axon degeneration in the aged somatosensory cortex (A) Example of a cortical axon at the pre-lesion time-point, with the injury site indicated by a star. (B) The same axon at 6 h post-lesion, showing the fragmentation of the distal portion. (C) At 1 day post-lesion, the distal portion of the axon is fully degenerated, indicated by the white dotted line. Red dotted line: crossing vessel causing reduced signal intensity.

This suggests that retraction rates in aged axons are largely consistent with those observed in young non-regenerating axons.

### Axon degeneration in the aged brain is comparable to the young brain

To study the degeneration dynamics of EPB-rich axons in the aged mouse cortex, imaging was conducted at multiple intervals post-lesion until at least 50% of the distal axon portion had degenerated. Acute Axonal Degeneration (AAD) is characterized by the rapid fragmentation of axons (within minutes) and has been observed in the spinal cord,^25^ and optic nerve.^26^ However, consistent with prior studies on the cortex of young adult mice,^11^^,^^12^^,^^27^ AAD was not observed in our experiments in the aged mouse cortex (*n* = 14). Approximately 85% (12 out of 14) of distal axons underwent complete degeneration by 1-day post-lesion, with all showing at least 50% degeneration within the first 6 h (Figure 3). The remaining 15% degenerated fully by day 2. For axons where the fragmentation process of the distal portion (Wallerian Degeneration, WD) was captured (*n* = 6), the onset of fragmentation occurred between 1 and 3 h post-lesion. However, due to the absence of short-interval time-points in our study, a quantitative comparison to young adult axons was not feasible. Qualitatively, the degeneration dynamics appear to be like those observed in young adult cortical axons in previous studies.^11^^,^^12^^,^^27^

### Increased synaptic dynamics following injury in the aged brain

**Figure 4:**
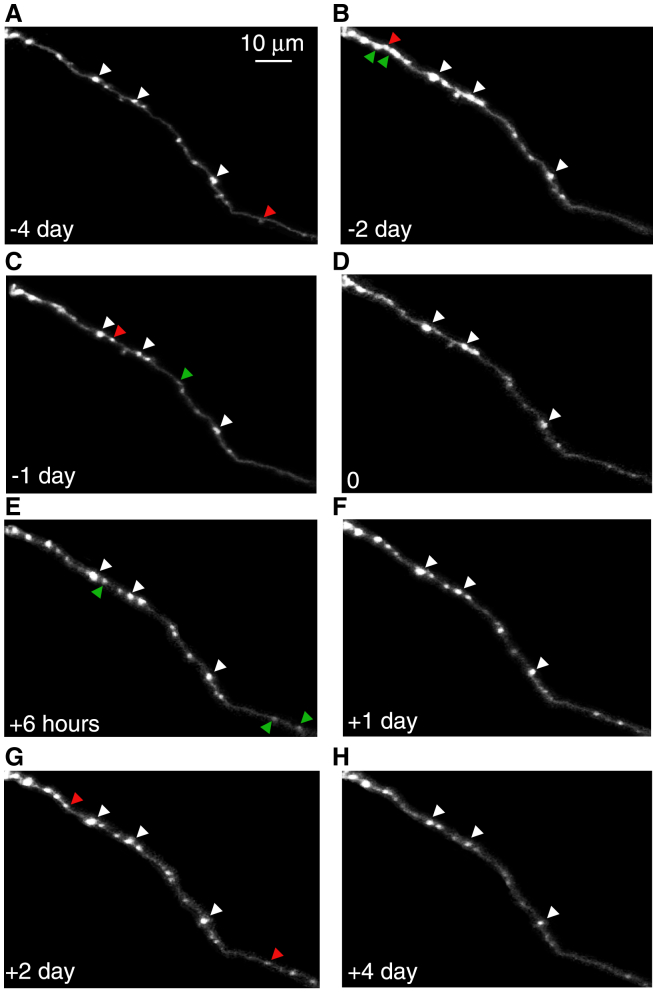
Two-photon *in vivo* imaging of EPB turnover in the aged mouse cortex (A–D) Pre-lesion EPB dynamics. (E–H) Post-lesion EPB dynamics. White arrows, stable EPBs Green arrows, new EPBs; Red arrows, eliminated EPBs.

To study synaptic changes over time in the aged, injured cortex, we imaged 8 axons (n = 277 EPBs) over 8 time-points before and after laser-mediated axotomy (−4 days, −2 days, −1 day, 0, +6 h, +1 day, +2 days, +4 days). An example of typical bouton addition and elimination over time is shown in Figure 4. This methodology allowed us to capture dynamic changes in presynaptic boutons in response to injury and provides insights into the plasticity of the aged cortex post-lesion.

**Figure 5:**
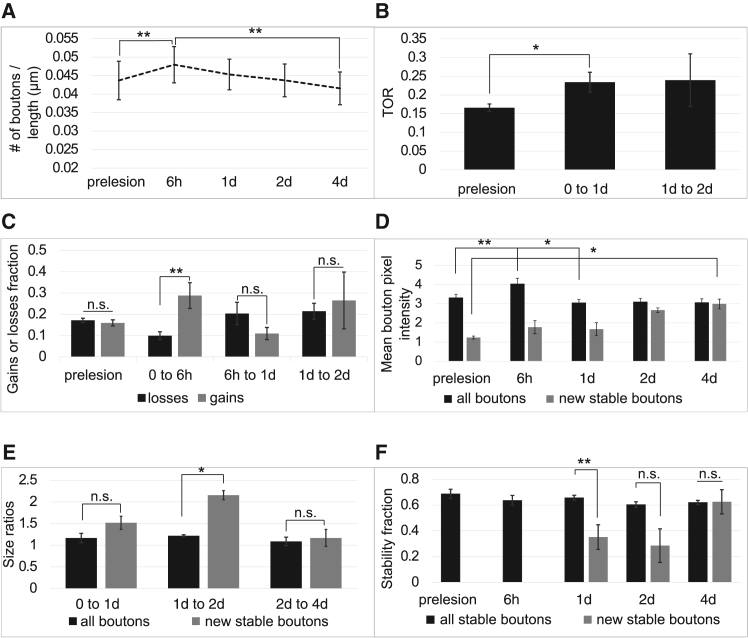
Quantification of EPB dynamics before and after injury in the aged somatosensory cortex (A) Density of boutons over time. (B) The TOR of 1 day time intervals. Prelesion is the average across the [-2 days to -1 day] and the [-1 day to 0] time-intervals. (C) Gains and losses fraction for different time-intervals. Prelesion is the average across all prelesion time intervals, including [-4 days to -2 days], [-2 days to -1 day], and [-1 day to 0]. (D) Mean bouton pixel intensity across all time-points. Prelesion is the average across all prelesion time-points, including: -4 days, -2 days, -1 day, and 0. (E) The size ratios of boutons between 2 time-points. (F) The stability fraction of all stable boutons, and new boutons that have stabilized following injury. Prelesion is the average across all prelesion time-points, including: -4 days, -2 days, -1 day, and 0. TOR, turnover rate; ∗, *p <* 0.05; ∗∗, *p <* 0.01; Error bars, SEM.

EPB density increased from 0.043 ± 0.012 (pre-lesion) to 0.048 ± 0.012 at +6 h (n = 8). The overall effect across timepoints was significant (Friedman test, *p* = 0.0024). Post-hoc Wilcoxon signed-rank test with Bonferroni correction showed a significant increase pre-lesion versus +6 h (adjusted *p* = 0.0078); Figure 5A, and a return to baseline by +4 days post-lesion (0.043 ± 0.016; +6 h vs +4 d, adjusted *p* = 0.0078). We next determined the TOR, comparing the pre-lesion time-intervals (0.16 ± 0.028), and the [0 to 1day] time-interval (0.23 ± 0.07), and found a significant increase (*n* = 8 axons, *p* = 0.039, Wilcoxon signed-rank). Furthermore, when examining whether the increase in TOR was due to increase in gains or losses (Figures 5C and 5D), we found no significant difference between gains and losses over one day, but there was a significant increase in total number of gains (0.29 ± 0.17) vs. losses (0.1 ± 0.05) in the [0 to 6h] time-interval (*p* = 0.0028, Man-Whitney U; Figure 5C). This indicates an immediate response to injury, reflected by a net increase in bouton number (Figures 5A–5C), driven by a higher fraction of gained compared to lost boutons. Both gains and losses go back to baseline rates by 2 days post-lesion, i.e., there is no net EPB gain or loss over time (Figures 5A–5C).

We next studied the relative size changes of boutons pre- and post-lesion (Figures 5D and 5E), a measure of synaptic strength.^19^ We plotted the normalized mean bouton intensity across all time-points, and the size ratios between two time-points. We found an increase at 6 h, but it was not maintained over a longer time (bouton size at pre-lesion = 3.30 ± 0.52, 6 h = 4.03 ± 0.81, at 2 days = 3.04 ± 0.51, *p* = 0.0078, Wilcoxon signed rank with Bonferroni correction).

**Figure 6:**
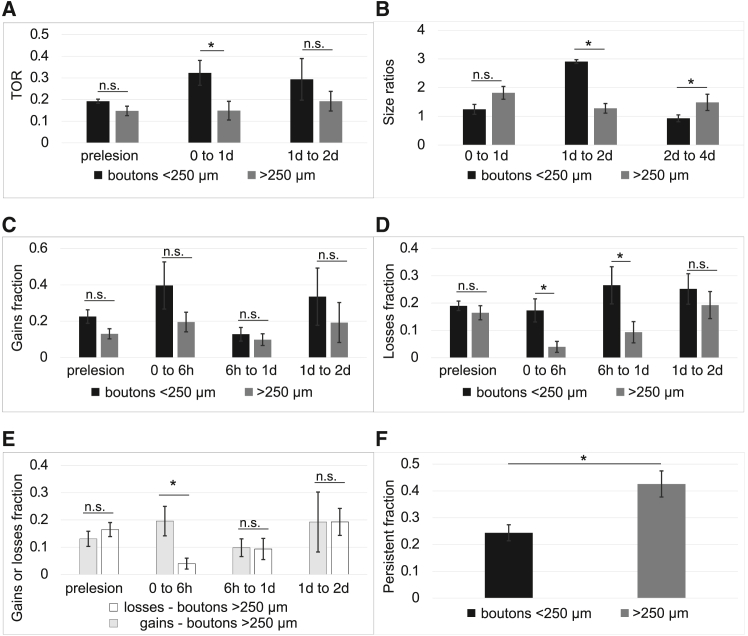
Quantification of EPBs close and far from the injury site We categorized EPBs into two groups: boutons close to the injury site (<250 μm) and far from the injury site (>250 μm). (A) Turnover rate (TOR) over 1-day time intervals. Pre-lesion values represent the average across the [-2 days to −1 day] and [-1 day to 0] time intervals. (B) Size ratios of boutons between two consecutive time points. (C–E) Gains and losses fractions for different time intervals, categorized into boutons close to and far from the injury site. Pre-lesion values represent the average across all pre-lesion time intervals, including [-4 days to −2 days], [-2 days to −1 day], and [-1 day to 0]. (F) Persistent fraction of boutons close to and far from the injury site. Persistent boutons were those present in all imaging sessions. TOR, turnover rate; *p* < 0.05; Error bars, SEM.

However, new boutons that became stable increased their size until they reached average bouton size (new bouton size at pre-lesion = 1.23 ± 0.19, at 4 days = 3.00 ± 0.67; *p* = 0.0156, Wilcoxon signed rank with Bonferroni correction), possibly indicating a re-wiring mechanism. Overall, there was also no difference in size ratios of all boutons when looking at 1-day intervals, suggesting that there is a homoeostatic mechanism which keeps the overall output of the neuron constant over a longer time scale. We also studied the size ratios of new and stable boutons that appear after injury. Stable boutons are defined as those being present for at least two consecutive sessions. New boutons that are also stable increase more compared to all boutons in the [1 day to 2 days] time-interval (1.21 ± 0.27 all boutons vs. 2.15 ± 0.26 new stable boutons at 1 or 2 *d*ays; *p* = 0.014, Mann-Whitney U), and go back to baseline by day 4. When comparing the stability fraction of all boutons and new boutons after injury, we find that they are comparable (0.62 ± 0.13 all boutons vs. 0.62 ± 0.24 new boutons at 4 *d*ays; *p* = 0.51, Mann-Whitney U; Figure 6F), suggesting that new boutons post-injury are just as likely to become stable as all other boutons.

Overall, we found that after injury, there is an increase in synaptic dynamics in which boutons are gained and lost soon after injury, and these new boutons become stable and increase their size more than pre-existing boutons. This might indicate an attempt to compensate for the loss of synapses that was caused by the injury.

We next investigated potential differences in the dynamics of boutons based on their proximity to the injury site (Figure 6). Boutons located within 250 μm of the lesion were classified as “close boutons” (*EPB*_*close*_), while those positioned farther away than 250 μm were categorized as “distant boutons” (*EPB*_*far*_*).* When looking at turnover rates of close vs. far boutons (number of *EPB*_*close*_ = 134, number of *EPB*_*far*_ = 143), we observed that the portion of the axon closer to the injury site was affected to a greater extent than the portion that was farther away. This is indicated by an overall higher TOR at [0 to 1day] time-interval (0.32 *±* 0.16 *EPB*_*close*_ vs. 0.14 *±* 0.12 *EPB*_*far*_, *p* = 0.039, Mann-Whitney U), and that there were more non-persistent boutons (0.75 *±* 0.08 *EPB*_*close*_ vs. 0.57 *±* 0.13 *EPB*_*far*_, *p* = 0.019, Mann-Whitney U) and fewer persistent boutons (0.24 *±* 0.08 *EPB*_*close*_ vs. 0.42 *±* 0.13 *EPB*_*far*_; *p* = 0.013, Mann-Whitney U). Overall, boutons closer to the injury site (*EPB*_*close*_) exhibited higher losses compared to those farther away (*EPB*_*far*_) at [0–6 h] (0.17 ± 0.12 *EPB*_*close*_ vs. 0.04 ± 0.06 *EPB*_*far*_; *p* = 0.04, Mann-Whitney U) and [6 h–1 day] (0.27 ± 0.19 *EPB*_*close*_ vs. 0.09 ± 0.1 *EPB*_*far*_; *p* = 0.038, Mann-Whitney U). Additionally, while there was no significant difference between the fractions of gains and losses for *EPB*_*close*_ (*p* = 0.2, Mann-Whitney U), boutons far from the injury site exhibited more gains than losses (0.2 ± 0.15 gains vs. 0.04 ± 0.06 losses at [0–6 h]; *p* = 0.04, Mann-Whitney U).

Interestingly, stable boutons close to the injury site showed the greatest size increase at [1–2 days] (2.91 ± 1.02 *EPB*_*close*_ vs. 1.27 ± 0.45 *EPB*_*far*_; *p* = 0.031, Mann-Whitney U), whereas stable boutons far from the injury site exhibited a marked size increase at [2–4 days] (0.93 ± 0.15 *EPB*_*close*_ vs. 1.5 ± 0.33 *EPB*_*far*_; *p* = 0.031, Mann-Whitney U).

These findings suggest that boutons near the injury site are more dynamic after injury and experience greater losses. Conversely, boutons farther from the injury site are more stable and show growth over time, potentially reflecting an attempt to rewire or compensate for the loss of boutons caused by the injury.

### Computational modeling of injury responses in the aged versus young brain

**Figure 7:**
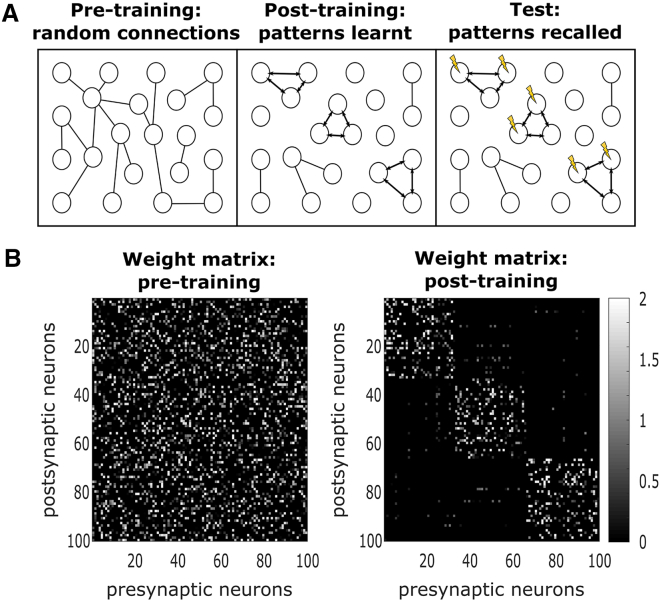
Modeling illustration and weight matrix evolution (A) Weights were initialized randomly according to connectivity observed in the somatosensory cortex (left). We trained “patterns” using external stimulation delivered to 3 sets of neurons (*N*_*pattern*_ = 33). The result was 3 highly connected patterns (middle). We use these “patterns” in an associate memory test to observe how well the network has learned from the training, by stimulating ∼60% of the neurons in each pattern (right), and computing a score based on the activation of the neurons that were not stimulated (i.e., “test neurons,” *N*_*test*_ = 13). (B) Example of randomly generated weight matrix pre- and post-training. The white squares represent the strength of the connection between a pre- and post-synaptic neuron. Black represents no connectivity between the neurons.

To investigate whether synaptic reorganization observed in the aged brain can promote recovery after injury, we employed a computational model. Unlike recurrent neural networks commonly used in machine learning, our model simulates biological neurons that exhibit spiking behavior and learn through a Hebbian learning rule (Tables 1, 2, 3, 4, 5, and 6). This approach is more biologically realistic compared to machine learning techniques such as backpropagation, which are often employed in artificial neural networks. The model is composed of 100 integrate and fire excitatory neurons^28^, which are recurrently connected (Figure 7B), with a Hebbian learning rule. Our aim was to study 1) whether the changes in synaptic dynamics, such as TOR or regeneration, affect the capability for memory recall, 2) whether an injury impairs recall, and 3) whether recovery is possible following injury.

**Table 1:** Parameters for simulation

Parameters	Description	EPB	TB
***I**ext*_*r*_	Strength of external current stimulation in training	3 pA	3 pA
***I**ext*_*e*_	Strength of external current stimulation in testing	10 pA	10 pA
***r**ext*_*r*_	Rate of external current stimulation in training	400 Hz	500 Hz
***r**ext*_*e*_	Rate of external current stimulation in testing	270 Hz	270 Hz
***r***_*noise*_	Rate of noise stimulation	100 Hz	100 Hz
***N***_*pattern*_	Number of neurons in each pattern	33	33
***N***_*test*_	Number of test neurons	13	13
***Sim***_*train*_	Total training length	1800 ms	1200 ms
***Sim***_*test*_	Total testing length	2610 ms	2610 ms
***sim***_*train*_	Training external current stimulation length	50 ms	50 ms
***sim***_*test*_	Testing external current stimulation length	270 ms	270 ms
***g***_*train*_	Time between stimulations in training	100 ms	50 ms
***g***_*test*_	Time between stimulations in testing	20 ms	20 ms
***reps***_*train*_	Repetitions of stimulations in training	4	4
***reps***_*test*_	Repetitions of stimulations in testing	3	3
*dt*	Time-step	0.1 ms	0.1 ms

These parameters were fitted to induce the high connectivity in the patterns during training, to have enough stimulation to induce associative memory during testing, and also to ensure that firing rates remained at biological levels.

**Table 2:** Parameters for injury model

Parameters	Description	Aged	Young	Young
		EPB	EPB	TB
*Bloss*_*injury*_	Fraction boutons lost because of injury	0.34	0.34	0.34
*Bgain*_*i*1_	Fraction boutons gained after injury (stage 1)	0.188	0.025	0.26
*Bsize*_*i*1_	Fraction bouton size change after injury (stage 1)	1.22	1	1
*Bloss*_*i*2_	Fraction boutons lost after injury (stage 2)	0.188	0	0.1
*Bsize*_*i*2_	Fraction bouton size change after injury (stage 2)	0.82	1	1
*BsizeNew*_*i*2_	Fraction new bouton size change after injury (stage 2)	2.41	1	1

Parameters for the aged condition were derived from the experiments described in this paper, while the parameters for young EPB and TB were obtained from.^11^^,^^12^ B, bouton

**Table 3:** Parameters for membrane neural dynamics

Parameters	Description	EPB	TB
***T***_*m*_	Membrane time-constant = RC	28 ms	28 ms
***u***_*rest*_	Reset voltage	*−*72 mV	*−*66.8 mV
*θ*	Threshold voltage	*−*38 mV	*−*40.2 mV
*R*	Membrane resistance	188 *M* Ω	277 *M* Ω

All parameters were taken from Lefort et al.^29^

**Table 4:** Hebbian learning rule *F*

	*ν*_*j*_ < (*ν*_*j*_)	*ν*_*j*_ > (*ν*_*j*_)
*ν*_*i*_ < *(ν*_*i*_*)*	nothing	^*↓ w*^*ij*
*ν*_*i*_ > *(ν*_*i*_*)*	^*↓ w*^*ij*	^*↑ w*^*ij*

*w*_*ij*_ is the synaptic strength between neurons *i, j*; *ν*_*i*_*, ν*_*j*_ are presynaptic and postsynaptic firing rates, respectively; *(ν*_*i*_*)* and *(ν*_*j*_*)* are the thresholds for firing; ^*↑*^, increase connection; ^*↓*^, decrease connection.

**Table 5:** Parameters for synaptic plasticity

Parameters	Description	Values
***β***	Hebbian learning rate	0.01 *≤β ≤* 0.05 pA
***τ***_*ν*_	Time constant of low-pass filtered spike train	20 ms
***τ***_*s*_	Spike time-constant	0.01 ms
***t***_*w*_	Time-window for firing rate	10 ms
***θ***_*synaptic*_	Weight threshold for synapse gain or loss	0.4 mV

The β parameter was individually fitted for each condition (aged EPB, young EPB, and young TB). All other parameters remained consistent across conditions and were optimized to facilitate learning during the training phase.

**Table 6:** Parameters for network graph

Parameters	Description	EPB	TB
***w***_*max*_	Upper weight boundary in learning	1.28 mV^29^^,^^30^	0.64 mV^29^
***w***_*min*_	Lower weight boundary in learning	0 mV	0 mV
***w***_*init*_	Weights are initialised between 0 to this value	0.64 mV	0.64 mV
***P***_*con*_	Probability of connections between neurons	0.23^29^	0.23^29^
*N*	Number of neurons in the network	100	100

**Figure 8:**
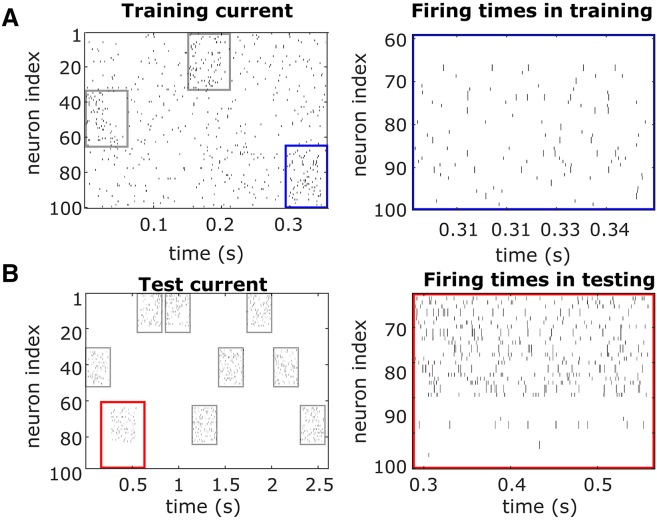
Training and testing in the computational model We performed our modeling simulation using a recurrent neural network using the Integrate and Fire model with Hebbian plasticity. (A) An example of a 1/4 training simulation. We trained “patterns” using external stimulation delivered, repstrain = 4 times (the figure shows only 1 rep), to 3 sets of neurons (*Npattern* = 33), for a total simulation length of Simtrain = 1.8s. The result was 3 highly connected patterns. Average firing rates, 10 Hz and 26 Hz (EPBs vs. TBs); Maximum firing rates, 80 Hz and 148 Hz (EPBs vs. TBs). (B) An example of an entire testing simulation (Simtest = 2.61s). Each pattern was tested repstest = 3 times. We observe how well the network has learned from the training, by stimulating 60% of the neurons in each pattern, and computing a score based on the activation of the neurons that were not stimulated (i.e., “test neurons,” Ntest = 13). Average firing rates, 57 Hz and 50 Hz (EPBs vs. TBs); Maximum firing rates, 184 Hz and 165 Hz (EPBs vs. TBs). Black lines, neuron spikes or firing times; Gray boxes, stimulation times; Blue box, location for firing times in training; Red box, location for firing times in testing.

This model was designed to investigate how alterations in synaptic dynamics influence memory storage. To simulate memory storage in the cortex, the model was trained to develop “patterns,” representing distinct encoded memories (Table 1). These patterns are groups of highly interconnected neurons (Figure 7B) that evolve during training through repeated stimulation with external current (an example of a training simulation is shown in Figure 8A).

During training, the strength of connections between neurons was captured in a weight matrix W. We observed significant potentiation among neurons within the same pattern (Figure 7B). Additionally, sparse noise, in the form of external current, was applied during training (Figure 8A, left).

To evaluate the effectiveness of memory storage, we conducted an associative memory task after training (Figure 7A, right). During testing, approximately 60% of the neurons within each pattern (20 out of 33) were stimulated with high external current (Figure 8B, left). Recall performance was assessed by determining whether the remaining unstimulated neurons in the pattern (13 out of 33) were activated by the test current (Figure 8B, right).

Finally, in the injury model, we investigated how young and aged brains respond to injury using parameters derived from the injury experiments. Performance comparisons were made between aged EPB and young EPB conditions, as well as between aged EPB and young TB conditions, to assess the potential for recovery following injury.

### Baseline model results

From our baseline simulations (i.e., training patterns without applying injury), we observed several interesting properties. Notably, the analysis of both our EPB and TB simulations yielded comparable results. Our initial focus was on examining how the learning rate (β) influences the turnover rate (TOR) in the model. We found a positive correlation between β and TOR (*n* = 50, *r* = 0.97, *p* < 3*e*^*−5*^ Pearson’s correlation), and error (*n* = 50, *r* = 0.81, *p* < 3*e*^*−5*^, Pearson’s correlation).

Higher TOR, representative of axons in the aged brain, is positively correlated with increased error in the memory task (*n* = 50, *r* = 0.84, *p* < 3*e*^*−5*^, Pearson’s correlation). This indicates a decline in performance with higher TOR, consistent with experimental findings in aged brains.^19^ This decline may occur because while higher learning rates facilitate the faster formation of connections, they also promote faster loss (i.e., forgetting), leading to reduced stability over time. Given the presence of noise between training stimulations, higher learning rates likely contribute to the rapid forgetting of patterns. This is further supported by the observation that higher TOR is inversely correlated with connectivity (*n* = 50, *r* = 0.94, *p* < 3*e*^*−5*^, Pearson’s correlation).

**Figure 9:**
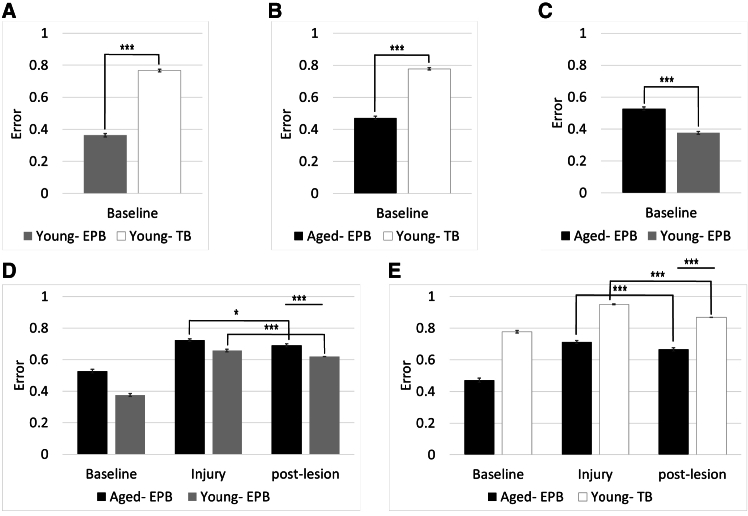
Modeling results (A–C) Baseline performance. EPBs are significantly better than TBs in performing a memory task (A and B). In addition, young EPBs have more optimal dynamics for storing memory, than aged EPBs (C). (D and E) Post-injury performance. All conditions showed a significant increase in error following injury.

We next examined the performance of models representing young and aged brains under baseline conditions (i.e., without injury; Figures 9A–9C). Parameters for aged EPB, young EPB, and young TB conditions were fitted (see STAR Methods for details), and memory task performance was evaluated post-training. Each simulation was repeated three times, and averages are reported.

We found that EPBs consistently outperformed TBs in the memory task, demonstrating significantly better performance (Figure 9A; 0.47 ± 0.09 error - young EPBs, vs. 0.77 ± 0.05 error - young TBs, *n* = 50*, p* = 7*e*^*−*^,^18^ Mann-Whitney U). This finding aligns with experimental studies suggesting that EPBs are specialized for encoding long-term memories due to their larger size and greater stability. In contrast, TBs are believed to play a supporting role, characterized by their smaller size and more dynamic nature (see Petrof and Sherman^30^ for a review of the topic). Additionally, young EPBs exhibited more optimal dynamics for memory storage compared to aged EPBs (Figure 9C; 0.37 ± 0.06 error - young EPBs; vs. 0.52 ± 0.02 error - aged EPBs, *n* = 50*, repeats* = 3*, p* < 3*e*^*−5*^, Mann-Whitney U). This is supported by results from prior work, which found that there is reduced performance in cognitive tasks in the aged brain.^19^

### Post-injury model results

**Figure 10:**
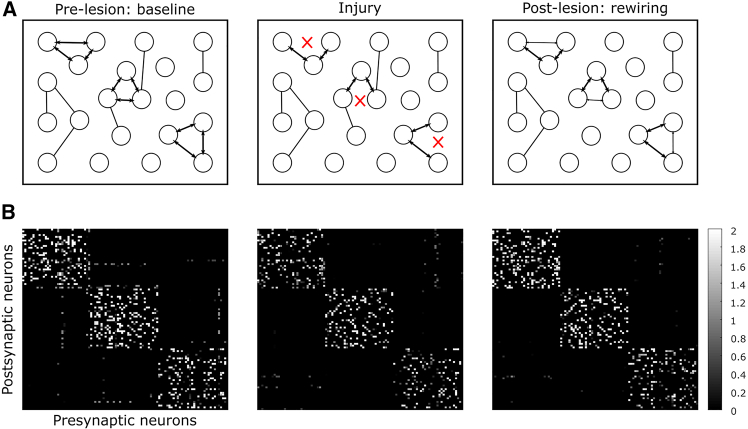
Injury model (A) The experiment tested parameters derived from aging and injury results. Left: A network was trained with parameters from the somatosensory mouse cortex, fitting TOR values from this and prior studies. Middle: Connections were severed to model injury, reflecting experimental observations. Right: Synaptic turnover was simulated post-injury to emulate the aged cortex, and memory performance was tested at each stage. (B) Weight Matrix Example. An aged EPB weight matrix across stages. Baseline (left): Pre-injury configuration. Injury (middle): Severed connections. Post-Lesion (right): Post-turnover adjustments. Training and testing followed each stage to assess memory performance. TOR, turnover rate.

We next investigated how the model of the aged and young brain responds to injury, mimicking experimental conditions (see STAR Methods and Figure 10 for details on the injury model). The detailed parameters used for aged and young bouton dynamics are shown in Table 2. As expected, all conditions exhibited an increase in error following injury (Figures 9D and 9E). Our analysis revealed that young EPBs have a superior capacity for memory storage compared to aged EPBs. Additionally, EPBs generally displayed more optimal dynamics for storing memories than TBs, underscoring their greater efficiency and stability in memory-related processes.

Following injury, we observed a small but significant recovery in the aged EPB model across all simulations (Figures 9D and 9E). When compared to young EPBs, aged EPBs showed worse performance post-injury (0.69 ± 0.08 for aged EPBs vs. 0.62 ± 0.06 for young EPBs, *p* = 2 × 10^−6^, Mann-Whitney U). Notably, young EPBs demonstrated the best overall performance post-lesion compared to all other conditions, suggesting that the dynamics of young EPBs are better suited for recovery after injury than those of aged EPBs.

In the young TB condition (Figure 9E), we observed the highest recovery from injury (0.86 ± 0.03 vs. 0.66 ± 0.08, young TBs vs. aged EPBs, *p* < 3*e*^*−5*^, Mann-Whitney U), with an error reduction of 0.08, bringing it closer to baseline performance than any other condition. Specifically, the young TBs were only 0.09 worse than baseline, whereas the aged EPBs were 0.19 worse than baseline. However, despite better recovery through regeneration, young TBs still exhibited significantly higher error rates compared to both young and aged EPBs, emphasizing that TBs are inherently less efficient at storing memory than EPBs.

In summary, our modeling simulations revealed distinct responses to injury in young and aged brains. While the young brain demonstrates the greatest recovery potential through regeneration, the aged brain can still achieve a small but significant recovery via synaptic re-wiring.

## Discussion

Our study sheds light on the critical role of aging in modulating axon regeneration and synaptic remodeling following cortical injury, advancing our understanding of the cellular and synaptic barriers that hinder neural recovery in the aged brain.

We used *in vivo* two-photon imaging through a cranial window on the aged somatosensory cortex to examine the response of axons to localized microlesions for a period of up to 3 months post-lesion. Notably, EPB-rich axons demonstrated increased synaptic dynamics immediately after injury, which subsequently returned to baseline levels—potentially reflecting a re-wiring mechanism in response to injury. Our modeling results indicate that transient synaptic disruptions within the first 24 h post-lesion, particularly near the injury site, are sufficient to destabilize attractor dynamics in associative networks, providing a potential mechanistic link between acute structural loss and longer-term impairments in memory storage and retrieval.

We also assessed axon degeneration, retraction, and regeneration *in vivo*. By the first day post-lesion, the degeneration of the distal portion was completed in 85% of cases, with 71% of axons showing at least 80% degeneration (Figure 3). The degeneration dynamics observed were qualitatively similar to those seen in the young adult cortex,^27^ further supporting the robustness of these findings. Quantitative analysis was not feasible due to insufficient comparable time points. However, we observed that the retraction of the proximal stump followed a biphasic pattern (Figure 2), with an acute phase occurring within the first day and a chronic phase extending up to three months post-lesion. These dynamics were consistent with retraction rates reported for non-growing axons in the young adult cortex^11^^,^^12^ (*n* = 9 aged, *n* = 53 young).

Interestingly, we observed no regeneration in aged EPB-rich axons, compared to a 20% regeneration rate in young adult axons from previous studies. These earlier findings demonstrated that 20% of EPB-rich axons from layers 2/3/5 and the thalamus in young adult mice (2–3 months old) regenerated over days and weeks. While it is possible that other cell types might exhibit higher regenerative capacity^12^ in the aged brain, our results align with the expectation of impaired axon regeneration in aged brains, consistent with their diminished ability to recover from disease and injury.^6^^,^^7^^,^^13^^,^^31^ These observations provide a detailed view of how axons respond to injury and contribute to understanding the mechanisms underlying neural repair or maladaptation.

### Extrinsic and intrinsic mechanisms for impaired regeneration in the aged brain

The aim of this study was to determine whether there are differences between the response of aged and young adult cortical axons to laser mediated microlesions. Because it has been shown previously that L2/3/5 and thalamocortical (TCA) EPB-rich axons are most affected by aging, we restricted this study to these axons. The rate of cortical axon regeneration in the brain is influenced by a combination of intrinsic and extrinsic factors. For extrinsic factors, one hypothesis for impaired axon regeneration is that the remaining distal axon segment might obstruct the path for regrowth. However, in this case, it is unlikely that slowed degeneration is the primary cause of reduced regeneration, as the degeneration rate in the aged cortex (Figure 3) appears comparable to that observed in young adult mice.^11^^,^^12^^,^^27^ Another extrinsic component that might influence regeneration is the presence of glial cells^32^^,^^33^, such as astrocytes, microglia, and glial scar. Currently, there is some controversy about whether the glial scar impedes or aids regeneration. For instance, while astrocytic scar was long thought to have a detrimental effect on axon regrowth, a recent study provides direct evidence for improved axon regeneration associated with astrocytic scar.^34^ This is also supported by a study in the cortex, demonstrating serotonergic axonal growth in the presence of a glial scar environment.^35^ However, since laser-mediated microlesions have consistently reported no significant glial scar formation,^11^^,^^12^ this is unlikely to have affected regeneration in this case. Microglia cells could also be a contributing factor for axon regeneration. Several different studies reported that microglia have age-related alterations^36^ and these alterations might cause a dysregulated response to injury.^37^ Their role in injury might be to clear debris remaining from the distal portion of the axon.^16^

Another possibility for impaired regeneration involves intrinsic mechanisms. While we lack direct evidence pointing to intrinsic factors, our observations reveal that the retraction rate of the proximal axon stump is similar to that of non-regenerating axons in young mice (Figure 2), suggesting a potential intrinsic limitation in the regenerative capacity. Increased rate and magnitude of retraction have been inversely correlated with growth in juvenile and young adult mice.^11^^,^^12^ A similar response was shown in another study, in which serotonergic neurons that lacked retraction had enhanced regrowth following injury, even in the presence of a glial scar.^35^ This suggests that axons with reduced retraction are more likely to regenerate, highlighting a connection to the intrinsic response of axons to injury. As axonal retraction did not differ significantly between young and aged mice (Figure 2I), the impaired regeneration observed in aged animals is unlikely to result from differences in initial injury response, but instead suggests intrinsic, age-related deficits in regenerative capacity. Additionally, age-related intrinsic changes in axonal biology could further contribute to impaired regeneration, potentially reducing the regenerative capacity of neurons over time. For example, growth cone generation has been suggested to be important for axon regeneration.^38^ In the cortex, a full growth cone is usually not present following injury, but instead, we observe a small bulbous axonal stump^11^^,^^12^ (Figure 2). Molecular regulators of axon growth, such as GAP-43,^39^ PTEN, and others,^40^ could also affect regeneration capacity following injury. It will therefore be important, in future work, to identify which intrinsic factors might cause the reduced regeneration in aged mice, as they could be potential targets for therapeutic intervention or for improving regeneration.

### Distinguishing synaptic remodeling from injury-induced axonal swelling

A key consideration when interpreting early synaptic changes following axonal injury is the potential for confounding structural alterations associated with acute axonal degeneration, such as transient membrane varicosities or blebbing at the proximal stump. These features, often driven by calcium influx and cytoskeletal breakdown (e.g., calpain-mediated spectrin degradation), have been well described in prior studies.^41^^,^^42^^,^^43^^,^^44^^,^^45^^,^^46^^,^^47^^,^^48^

However, in our analysis, we specifically excluded the immediate time points from quantification to avoid such confounds and instead focused on EPBs after at least 6 h post-lesion.

Importantly, we classified EPBs based on their distance from the lesion site and observed distinct patterns of turnover that varied with proximity. We found that boutons closer to the lesion (<250 μm) showed significantly higher turnover, greater losses at early time points (Figures 6A and 6D, 0–6 h and 6 h–1 day), and less persistence (Figure 6F), suggesting that proximity to injury strongly influences synaptic stability. In contrast, boutons farther from the lesion (>250 μm) were more stable (Figure 6F) and exhibited a delayed growth response (Figure 6B, 2d–4d), suggesting possible compensatory rewiring.

The temporal and spatial characteristics of these EPBs are not consistent with transient varicosities, which typically arise within seconds, are uniformly spaced, and resolve without exhibiting structured turnover dynamics within minutes, or they may be accompanied by large swellings with axonal thinning.^41^^,^^42^^,^^43^^,^^44^^,^^45^^,^^47^ Furthermore, previous correlative 2-photon and serial electron microscopy analyses have demonstrated that axotomy-induced boutons form ultrastructurally mature synapses, complete with synaptic vesicles, active zones, and dendritic contacts.^11^^,^^39^ Together, these observations argue that the synaptic dynamics reported here reflect injury-induced plasticity rather than pathological swelling, underscoring the importance of temporally and spatially resolved analysis when interpreting synaptic responses to axonal injury.

### Synaptic dynamics following injury in the aged brain - a maladaptive or compensatory mechanism?

The mechanism behind re-wiring in the aged brain post-injury remains unclear. However, it may represent an attempt to compensate for synaptic boutons lost on the distal portion of the axon. This contrasts with the young brain, where axon regeneration is the predominant response to injury. This adaptive re-wiring could reflect an alternative repair strategy in aging neural circuits, potentially influenced by reduced regenerative capacity or changes in synaptic dynamics with age. There are two prevailing theories regarding the shift in synaptic dynamics following injury: either the increased synaptic turnover represents a compensatory mechanism aimed at functional recovery, or it reflects a maladaptive response to injury, potentially indicative of a dysfunctional state. The latter suggests that heightened synaptic remodeling might not effectively restore connectivity but instead exacerbate instability in neural circuits, particularly in the aging brain. Understanding whether these changes are adaptive or maladaptive is critical for designing targeted interventions to promote recovery. Further investigation is needed to clarify this dichotomy and its implications for brain repair mechanisms. For instance, increasing levels of TOR are consistently reported in both boutons and spines (with spines having higher TOR than boutons^49^) with cognitive impairment,^19^^,^^50^ injury,^11^^,^^12^ aging,^19^^,^^20^ and with sensory deprivation.^51^ A study examining bouton dynamics following the induction of Long-Term Depression (LTD) found a decrease in bouton size, and an increase in bouton TOR, indicating that decrease in connectivity is associated with increased bouton TOR.^52^ Indeed, we also find in our baseline modeling simulations that higher levels of TOR are associated with decreased performance in a memory task, both in EPB and TB-like axons. However, since the increased synaptic dynamics in response to injury were short lived and returned to baseline levels by day 4 post-lesion, it is unlikely that this change is maladaptive in the long term. Together, the elevated baseline synaptic turnover rate (TOR) in aged cortex and its transient increase following injury may exacerbate synaptic instability, disrupting the balance between plasticity and stability that is critical for effective circuit memory recovery.^3^^,^^53^^,^^54^ An important direction for future studies will focus on dissecting the interplay between injury-induced and age-related synaptic remodeling mechanisms.

Alternatively, it is possible that increased synaptic dynamics might be a mechanism of ensuring efficient learning even in old age^55^ or to compensate for the lack of regeneration observed in aged mice in this study. This theory is supported by several pieces of evidence. In our data, for instance, the net gain of boutons and size increase at 6 h post-lesion (Figure 5) might be an attempt to compensate for the boutons lost on the distal side of the axon by forming new connections with neighboring neurons or reinforcing existing connections. While there is a loss of boutons at 4 days post-lesion (with total bouton densities returning to baseline levels), many (60%, Figure 5F) of the newly formed boutons are kept. Indeed, many of the new boutons increase their size more than all other boutons post-lesion (Figure 5E), and also become stable (Figure 5F). This might indicate a pruning mechanism, in which the network attempts to compensate for lost boutons and/or impaired regeneration by optimising the available resources. To compensate for this, aged axons might rely on increased synaptic plasticity to cope with injury. Further evidence for re-wiring was shown by separating boutons into those close and far from the injury site (Figure 6). Boutons close to the injury site were affected the most by the injury, having higher rates of TOR (with higher losses), and lower persistence. From the quantitative analysis, we observed that while more boutons that are close to the injury sites are lost, more boutons far from the injury site are gained at 6h post-lesion. It is possible that this is due to a pruning mechanism in which boutons that are less necessary after injury are lost (or alternatively are removed because of the inhibitory environment, or microglia^56^), and new boutons are formed to compensate for that loss, for continued effective coding. Post-lesion re-wiring might occur due to the enhanced excitability of pre-synaptic neurons onto the injured neuron,^57^ which might aid recovery and maintenance of homeostasis following an injury. In addition, it is likely that other structural or functional changes in nearby neurons occur to aid recovery, which we cannot observe with the current techniques, since we only imaged the injured axon, and we have not measured the electrophysiological properties of the injured axon or nearby neurons. Since we only make a small, minor lesion, significant re-wiring or regeneration may not be necessary for complete recovery from injury, as neurons are highly connected in the cortex. Alternatively, complete recovery via axon regeneration or synaptic re-organization might be unachievable in the cortex. Imaging both pre- and post-synaptic dynamics after injury would be an elegant way to confirm whether there are other mechanisms at play.^58^

### Partial recovery following injury in a computational model

Finally, we investigated whether the increased synaptic dynamics observed in this study can aid recovery from injury, using a computational model of recurrent excitatory integrate and fire neurons with Hebbian plasticity. We used intrinsic electrophysiogical parameters from the somatosensory cortex, and had the synaptic dynamics (TOR) fitted to the aged (this study, and Grillo et al.^19^) and the young brain.^11^^,^^12^^,^^19^ Although the somatosensory neurons studied here are not directly responsible for memory storage, primary sensory areas are increasingly recognized as contributing to memory-related processes such as sensorimotor learning and experience-dependent plasticity.^59^^,^^60^^,^^61^ In this context, we use “circuit memory” to describe a network’s ability to maintain or restore stable synaptic configurations over time, especially after injury. Our computational model does not simulate memory per se but quantifies how synaptic dynamics impact circuit stability—a prerequisite for persistent neural function. The heightened synaptic turnover observed post-injury, particularly in aged cortex, reflects greater network instability, potentially compromising the long-term maintenance of circuit function and memory-related processes. These early synaptic disruptions may interact with later, prolonged phases of cortical plasticity post-injury, potentially influencing the trajectory of circuit reorganization and the functional outcomes of recovery.

We compared a model containing parameters of EPB-rich axons from the aged brain to models based on a young brain with two different dynamics, using an associative memory task (in which neurons were trained before the injury is induced). One model was based on EPB-rich axons from the young brain, which regenerated in only 20% of cases. The second was based on TB-rich axons, which have a higher baseline TOR, and regenerate considerably more (55%). We found that in the aged brain, the synaptic re-wiring following injury allowed a small partial recovery (Figures 9D and 9E). Young EPB-rich axons recovered more after injury and had the best performance compared to other conditions (Figure 9D). Young TB-rich axons recovered the largest amount from injury compared to all other conditions, but still were significantly worse in the memory task than both young and aged EPBs following injury (Figure 9E). This suggests that, according to our model, younger brains have better memory recall (Figure 9C, also supported by experimental findings^19^), the young brain can partially recover by regenerating the injured axon, and the aged brain can partially recover from injury using synaptic re-organization (Figures 9D and 9E). One interpretation of these findings might be that the young and aged brains have different mechanisms to cope with injury, one by regenerating injured axons and the other by re-wiring synapses to compensate for the lack of regeneration. TB-rich axons in the young brain had the largest recovery, but still had worse performance in the memory task (Figure 9E), possibly because TBs have a more modulatory role, rather than being optimized to memory storage.^30^ It might be that the role of the regeneration of TB-rich axons following injury is to modulate other neurons, and thus would still have worse memory capacity than their EPB counterparts. In addition, since the recovery found was not enough to return to baseline levels of performance, it is possible that there are mechanisms other than the regeneration or synaptic reorganization of the injured axon, that can aid recovery from injury. For instance, since we do not image the neurons which are connected to the axon we injure, we do not know whether they are also affected by injury. It is possible that there are other functional and structural changes that might aid recovery in the nearby neurons. Our current study uses a simplified model and does not account for all the intricate factors involved in post-injury dynamics in the cortex. As such, we cannot predict how these dynamics would affect the entire brain. While this lies beyond the scope of this study, future work using a more complex and biologically representative model of neurons and injury could provide valuable insights into these dynamics.

### Conclusions

In this article, we found that:

1. The aged brain exhibits impaired regeneration, which is not attributed to slowed degeneration.
2. Following injury, synaptic re-organization occurs, characterized by boutons being gained and increasing in size, before eventually returning to baseline levels.
3. Our computational model suggests that synaptic re-organization in the aged brain may act as a compensatory mechanism for injury, whereas the young brain primarily recovers through regenerating injured axons. However, according to our model, both mechanisms only enable partial recovery, suggesting that full recovery from injury might be unachievable in the cortex.

### Limitations of the study

We assume that injury induced varicosities are bona fide synaptic sites; however, this might not be the case in all instances. To exclude injury-induced varicosities from our analysis, all imaging was performed from 6 h to several days post-injury, that is, well beyond the acute phase (seconds to minutes) during which transient injury-induced blebs typically form and resolve.^41^^,^^42^^,^^43^^,^^44^^,^^45^^,^^47^ Moreover, our group and others have extensively validated that axonal varicosities observed under similar imaging conditions correspond to mature, synapse-bearing *en passant* boutons using *in vivo* imaging combined with retrospective EM and fluorescence microscopy.^11^^,^^19^^,^^39^^,^^62^^,^^63^

The computational model also has several limitations and relies on assumptions, which are described in detail here.

We use a simplified model, which offers the advantage of requiring fewer parameters to fit. However, this approach has limitations in terms of how accurately it represents the complexity of real neural networks.

1. Integrate-and-fire neurons provide a simplified representation and do not fully capture the dynamics of real neurons, such as refractory periods, adaptation, or bursting behavior. Since they reset the voltage to a fixed value without retaining memory of prior spikes, these features are not inherently modeled.^28^ While these properties could be incorporated to enhance biological accuracy, doing so would significantly increase the model’s complexity and the number of parameters.
2. Rate-based Hebbian plasticity does not account for the precise pre- and post-synaptic spike timing dynamics seen in models such as Spike-Timing-Dependent Plasticity (STDP).^28^ However, STDP learning rules can introduce pathological behavior in balanced networks^64^ and are often challenging to configure effectively.
3. Our model focuses exclusively on excitatory neurons, which does not fully represent the complexity of the cortical network. While more sophisticated models incorporating both excitatory and inhibitory neurons with balanced network dynamics have been developed,^65^ these often require a larger number of free parameters. Since our animal experiments were conducted solely on excitatory neurons, we chose to model a network of excitatory neurons, as the behavior of inhibitory neurons after injury is currently unknown.
4. The time-scales in our model are in seconds, whereas the time-scales in animal experiments are measured in hours or days. To address this discrepancy, we computationally reduce the time scale, as simulating the model over such long experimental periods would be computationally prohibitive. However, by calculating TOR values as a ratio between simulations with different parameters, our model remains aligned with the experimentally observed values.
5. Since no experimental results were available for TB-rich axons in the aged brain, we were unable to run simulations for this condition. However, given that TB-rich axons exhibited higher regeneration in the young brain, it is plausible that some level of regeneration might also occur in the aged brain.
6. We perform simulations with a relatively small number of neurons (n = 100). This approach significantly reduces computational resource demands while still providing reliable results in our simulations.

In addition, we make several assumptions about the model.

1. The turnover rate between neuronal connections was used to model aging, as it represents one of the key effects of aging on structural plasticity.^19^^,^^20^
2. To observe the effects on populations of neurons, rather than single neurons, we configured our model as a group of randomly connected neurons. This approach differs from our animal experiments, where we image synapses on single neurons and study their dynamics over time in response to injury. In our model, each connection between two neurons represents a bouton or a synapse, similar to what is measured in the animal experiments. Specifically, the connection weight in our model corresponds to the size or efficacy of a synapse as imaged using two-photon microscopy.
3. We assume distinct biophysical properties for EPB- and TB-rich axons, as they originate from different areas within the cortex—specifically, from L2/3/5 and the thalamus, and L6, respectively).^62^
4. We assume that following injury, re-wiring occurs most optimally, where connections are added to the patterns, and removed from outside the patterns. This, however, might not be the case, so we perform a baseline where connections are added randomly. With random re-wiring, we found similar but slightly worse recovery from injury than when using optimal re-wiring (see supplemental information for results).

Overall, we aimed to keep the model as simple as possible to isolate the effects of synaptic dynamics observed in our experiments and others on memory function. We validated the model by varying dt to confirm its accuracy and stability (see the dt experiment in the supplemental information). Additionally, we incorporated as many parameters as possible from biological experiments to enhance the model’s biological plausibility.^11^^,^^12^^,^^19^

## Resource availability

### Lead contact

Further information and requests for resources and reagents should be directed to and will be fulfilled by the lead contact, Vincenzo De Paola (vincenzo.depaola@duke-nus.edu.sg; vincenzo@imperial.ac.uk).

### Materials availability

This study did not generate new unique reagents.

### Data and code availability

• Data: The datasets generated and analyzed during this study, including imaging data from multiphoton microscopy of axonal injury in aged and young mouse cortex, are available from the lead contact.
• Code: The custom scripts used for preprocessing, statistical analysis, and computational modeling of recurrent neural networks are available in GitHub at https://github.com/CherBass/ModellingAgingInjury. The repository includes documentation and instructions to reproduce the analyses and simulations.
• Other: Any additional information required to reanalyze the data reported in this article is available from the lead contact upon reasonable request.

## STAR★Methods

### Key resources table

**Table:** 

REAGENT or RESOURCE	SOURCE	IDENTIFIER
**Chemicals, peptides, and recombinant proteins**		

Ketamine Hydrochloride	Pfizer	Cat# NDC00409-2051-01
Xylazine	Akorn	Cat# NDC59399-110-20
Lidocaine	Sigma-Aldrich	Cat# L7757
Isoflurane	Piramal	Cat# NDC66794-017-25
Agarose	Sigma-Aldrich	Cat# A9539
Buprenorphine	Reckitt Benckiser	Cat# NDC12496-0757-1

**Deposited data**		

Simulation and analysis code	GitHub	https://github.com/CherBass/ModellingAgingInjury
Data	Zenodo	https://zenodo.org/records/17165722

**Experimental models: Organisms/strains**		

Thy1-GFP-M mouse	Jackson Laboratory	Cat# 007788; RRID: IMSR_JAX:007788

**Software and algorithms**		

MATLAB R2022a	MathWorks	RRID:SCR_001622
ImageJ	NIH	RRID:SCR_003070
SPSS Statistics	IBM	RRID:SCR_002865
Excel	Microsoft	RRID:SCR_016137
GIMP	GNU	RRID:SCR_003182
Inkscape	Inkscape Project	RRID:SCR_014479

**Other**		

Two-photon microscope	Bruker Ultima Investigator	–
Chameleon Ultra II laser	Coherent Inc.	–
Custom MATLAB scripts	This paper	Available upon request or https://github.com/CherBass/ModellingAgingInjury

### Experimental model and study participant details

For all the experiments, we used aged male mice (Thy-1-GFP-M; *n* = 12, *>* 2 years old with cytosolic GFP expression^11^^,^^19^) on C57BL/6 genetic background. As described previously^11^ animals were housed with littermates, in standard, individually ventilated caging and maintained in a 12-h light/dark cycle with free access to food and water. All experiments involving live animals were conducted by researchers holding a UK personal license and conducted in accordance with the Animals (Scientific Procedures) Act 1986 (UK) and associated guidelines.

### Method details

#### Surgical and imaging procedures

##### Cranial window surgery

Surgery was performed as described previously.^11^^,^^19^ For all imaging experiments, a cranial window was implanted overlying the somatosensory cortex under anesthesia (0.083 mg/g ketamine, 0.0078 mg/g xylazine). Intramuscular dexamethasone (0.02 mL at 4 mg/mL) was administered to reduce inflammatory response, and subcutaneous bupivacaine (1 mg/kg), a local anesthetic. The skull was exposed using a dental drill, and a few drops of lidocaine (1% solution) were applied on its surface prior to drilling. The glass coverslip that seals the window was placed directly over the dura and the bone edges, with a thin layer of agarose in between, and sealed with dental cement. Mice were given 2-3 weeks to recover and for the cranial window to clear, before the start of the imaging. Post-operative care was done by personal license holders, and trained staff at the animal house. Animals were monitored continuously for the first hour after surgery, daily until fully recovered (minimum 72 h), and then at least three times a week after that to ensure no adverse effects were seen. Post operation analgesia was given (e.g., buprenorphine) within 72 h post-surgery, as advised by the Named Veterinary Surgeon (NVS). A number of humane endpoints were observed, and mice were sacrificed by a schedule 1 method or non-schedule 1 method, if two or more of the following clinical signs were present: piloerection, hunched posture, reduced activity, increased docility or aggression, weight loss up to 20% of body weight, dehydration persisting for 24 h during fluid replacement therapy, altered respiration, or self-mutilation.

##### Multiphoton imaging

Imaging was performed similarly to Canty et al.^11^ with a few minor alterations, described below. A two-photon microscope equipped with a tunable Coherent Ti:Sapphire laser and Bruker software for image acquisition was used for all imaging experiments. Mice were anesthetized with Isoflurane, an inhalation anesthetic, and secured to a fixed support under the microscope. To prevent dehydration in the eyes, Lacri-lube (Allergan) was applied. To regulate body temperature (37°C) an underlying heat pad was used and rehydration administered with isotonic saline solution (i.p.) required during long imaging sessions. Depth of anesthesia was closely monitored by a video camera and regularly checking reflexes (toe pinch) and respiratory rates. An Olympus 4X lens with a 0.13 numerical aperture (NA) was used to identify characteristic blood vessel patterns, to relocate axons used in previous imaging sessions. An Olympus 40x with a 0.80 NA water-immersion objective was used to acquire the images. For optical zoom 1, images were typically 300 × 300 μm field of view, 512 x 512 pixels, 0.588 μm per pixel for *x, y* planes, and 3 μm per pixel for the *z-*plane. For optical zoom 4, images were typically 75 × 75 μm field of view, 512 x 512 pixels, 0.147 μm per pixel for *x, y* planes, and 1 μm per pixel for the *z* plane. A point spread function was characterised, for images with optical zoom 4, by a Full Width at Half Maximum (FWHM) values of 0.45 x 0.45, 2.5 μm (*x, y, z*)). Either a water repellent pen or Vaseline (pure Petroleum Jelly) was applied around the cranial window to stabilize the meniscus for the 40x objective. A pulsed 910 nm laser beam was used, never exceeding 70 mW on the back focal plane. Each imaging session typically lasted for 60–90 min, during which time up to 40 image stacks (1 μm step size for images that were analyzed) were collected. Typically, 1-2 non-overlapping axons were selected for lesioning in each animal. For synaptic analysis *in vivo*, we first identified long axonal stretches and then collected high-magnification images of the synapses along the axon shaft at 6 h, 1 day, 2 days, 4 days, weekly, and monthly intervals (Figure 1C). The identification of the same axons was done using anatomical reference points (e.g., vessels and bifurcation points).

##### Laser mediated micro-lesions

We used a Chameleon Ti:Sapphire, using a 9 μm diameter binary mask, ∼1000 mW at the back focal plane, 800 nm, and 30 μs dwell time to induce localised injuries in cortical layer 1. A total of 13 axon branches were lesioned across all imaging experiments and were observed up to 3 months post-lesion (*n* = 6 mice, *>* 2 years old). To standardize segment size for regeneration analysis, as done in previous studies, the reported axon segment lengths were measured along the disconnected visible axons from the lesion site, not from the neuronal cell body. The amount of distal axon removed was between ∼150 and 500 μm (on average 314 ± 111 μm; in case multiple endings were present, we considered the total length of the disconnected portion), leaving a minimum of 250 μm of the surviving axon for post-lesion analysis, as in previous experiments.^11^ Identified axons were severed by inducing a lesion using a single circular spot scan. Lesions were induced in the upper layers of the cortex, to a depth of 50 μm beneath the pial surface. Lesions were made as far apart as possible (minimum 300 μm). Cortical axons were imaged at variable intervals (Figure 1C) before and after lesion for as long as window clarity was preserved, or up to 3 months.

We followed the axons for at least two days post-lesion, and were able to confirm in all cases that the correct axons were injured. During one of the imaging sessions, overlapping low-magnification overviews of the labeled blood vessels were collected to help identification of axons in the fixed brain.

#### Synaptic and axonal analysis

##### Data analysis

The two-photon images were processed using a custom made, semi-automated MATLAB MathWorks package called EPBscore^66^ (synaptic remodeling), and ImageJ (length quantification, file conversions). For further bouton dynamic analysis, we used a MATLAB script to compute all discussed metrics. For bouton counting, we also used the bouton detection software from.^67^ Figures were prepared using MATLAB, Microsoft Office Suite, ImageJ, Gimp and Inkscape. Axons were classified by morphological criteria, as previously described.^62^ Only EPB-rich axons were monitored in the imaging experiments.

###### Axon degeneration

We calculated degeneration dynamics by measuring the total axon length (including both proximal and distal segments) and then determining the fraction of the remaining distal axon length at each available time point.

###### Axonal retraction

To calculate axon retraction, we measured the distance from the proximal tip to the lesion site at several intervals post-lesion. Growth was not visible in any axons that were imaged. We obtained measurements for 6 of the 7 axons that were lesioned, at days 0, 1, 2, 4, 11 and 32 post-lesion.

###### Axon regeneration

To calculate regeneration, we measured the distance grown from the proximal stump between two time-points. We defined attempted regrowth if the elongation exceeded twice the maximum noise measurement (that is, >6 μm; the maximum difference in measurements between fiducial points over repeated imaging sessions is 3 μm).

###### Axonal bouton quantification

EPB-rich axons were imaged and analyzed. EPB rich axons have cell bodies that are located in either of L2-3, 5, or the thalamus, mainly form axo-spinous synapses and are relatively stable in the adult brain.^62^ The bouton metrics reported in this paper were calculated in the following way.

1. Density of boutons over time was defined as: *Density* (*a*) = *n*_*a*_*/length*_*a*_ (μm), where *n*_*a*_ is the total number of boutons in session *a*. Pre-lesion refers to the average across the following time-points: -4 days, -2 days, -1 day, and 0.
2. EPBs near the injury site were selected based on their distance to the injury. EPBs classified as “close” were located within 250 μm of the injury site, while those classified as “far” were situated more than 250 μm away.
3. Turnover rate (TOR) between two imaging sessions a and b, is defined as (*nG* + *nL*)*/*(2*N*), where *nG* and *nL* are the numbers of bouton gains and losses respectively, and *N* is the total number of boutons in session a. The daily TOR pre-lesion are calculated by averaging across [-2 to -1day] and [-1 to 0 days] time-intervals.
4. Gains and losses fractions were defined as: *Gains*(*a, b*) = *nG*_*b−a*_*/N* and *Losses*(*a, b*) = *nL*_*b−a*_*/N*.
5. The size ratio (Δ*S*) of boutons between two imaging sessions a and b is defined as: Δ*S* = (*S*_*b*_)*/*(*S*_*a*_)), where *S*_*a*_ is the size of a bouton at session a, and *S*_*b*_ is the size of a bouton at session b. A ratio higher than 1 demonstrates an increase in size, whereas a ratio smaller than 1 demonstrates a decrease in size. We plotted the average size ratios between different time-points before and after injury. Pre-lesion refers to the average across the following time-points*:* -4 days, -2 days, -1 day, and 0.
6. Stable boutons were defined as boutons that were present for at least two consecutive imaging sessions during a specific session.
7. New boutons referred to boutons that appeared for the first time after the injury.
8. New and stable boutons were boutons that appeared post-injury and remained present for at least two consecutive sessions.
9. Persistent boutons were boutons that were present throughout all imaging sessions.
10. Destabilized boutons were those present during the first three sessions (for at least two consecutive sessions) but were completely lost before the last two imaging sessions.

##### Computational modeling

All computational modeling was carried out in MATLAB using a simple recurrent leaky integrate-and-fire network consisting of 100 excitatory neurons with Hebbian plasticity. We simulated a network of 100 neurons to capture essential aspects of circuit-level dynamics in a computationally efficient framework, consistent with prior studies that have used small networks to investigate fundamental neural mechanisms.^68^ This model was used to test whether the synaptic dynamics observed in the aged and young brain could facilitate recovery from injury. To assess general memory performance, we employed an associative memory task and evaluated the network under different conditions, including before and after injury, and in both the aged and young brain.

##### Simulation protocols

The model consisted of *N* excitatory neurons. Connections occurred with probability *P*_*con*_, and the strength of a connection from neuron *i* to neuron *j* was denoted by *W*_*ij*_ (Figure 7, left). We aimed to train 3 “patterns”, which are 3 groups of highly connected neurons (Figure 7, right). They were trained with *reps*_*train*_ = 4 repeated presentations of external current with randomised order (Figure 8A, Table 1). These trained patterns were then used to test performance in a memory task. We used an associate memory task to test recall, by stimulating a percentage (60%) of the neurons in each pattern, with high external current, as in the training (pattern neurons = *N*_*pattern*_, test neurons = *N*_*test*_). Performance was recorded using an error metric, which was calculated by counting the total number of spikes from neurons that were not directly stimulated (Figure 9B). Performance was compared across different conditions in both baseline and injury simulations. The total training time for the network was set to Simtrain = 1.8s, while the total testing time was Simtest = 2.61s. During both training and testing phases, external current with a rate of *r*_*Iextr*_ (for training) or *r*_*Iexte*_ Hz (for testing), and of strength *I*_*extr*_ (for training) or *I*_*exte*_ pA (for testing), was activated sequentially. The strength of the external current was equivalent to the sum of approximately 5 input neurons during training and approximately 15 input neurons during testing. This external current was applied for the duration of *sim*_*train*_ or *sim*_*test*_, with a gap of *g*_*train*_ or *g*_*test*_ between stimulus presentations. Neurons were targeted by each external stimulation with rate of *r*_*Iext*_ Hz, and by noise with rate of *r*_*noise*_. As a result, during training, firing rate was on average 10 Hz and 26 Hz (EPBs vs. TBs), comparable to firing frequencies in the somatosensory cortex (11 ± 0.82 Hz)^69^^,^^70^ with a maximum firing rate of 80 Hz and 148 Hz (EPBs vs. TBs) during the stimulation. In testing, the average firing rate was 57 Hz and 50 Hz (EPBs vs. TBs), and the maximum during the stimulation was 184 Hz and 165 Hz (EPBs vs. TBs). While this is a high firing rate in comparison to most living neurons, it does not surpass the highest firing rates observed in the somatosensory cortex (185 *±* 10 Hz).^69^^,^^71^

##### Metrics

To calculate the performance of each model, we used the error metric. To compute the error, during the test we counted all the test neurons that fired during each test simulation for each pattern. Neurons that fired at the correct time (i.e., during the test current stimulation), were counted as True Positives (TP), and those that fired at other times were counted as False Positives (FP). We used TPs to compute the Error (E) metric:

(Equation 1) $$E=1-\frac{TP}{{reps}_{test}}$$

where *reps*_*test*_ is the number of test stimulations per pattern.

##### Injury model

To model injury, we generated a weight matrix for each condition with parameters fitted (Figure 10; Table 2) using experimental data. We performed the following comparisons in our simulations: (1) Aged EPB-rich axons vs. young EPB-rich axons (*n* = 50, repeats = 3), and (2) Aged EPB-rich axons vs. young TB-rich axons (*n* = 50, repeats = 3, we fitted different biophysical values for TB-rich axons since they originate in L6, in contrast to EPB-rich axons which originate in L2/3/5 and thalamus). Since TB-rich axons exhibited greater regeneration compared to EPB-rich axons in the young brain, we anticipated improved recovery in this condition. For each condition we generated a weight matrix for each of the steps (Figure 10B). The steps include; baseline (i.e., pre-lesion), injury (i.e., boutons lost straight after injury), and post-lesion (i.e., re-wiring). At each step we performed an associate memory test, which is preceded by one repeat of a training simulation with Hebbian learning, aimed to mimic a more realistic dynamic environment as in the living brain. Specifically, we cut *Bloss*_*injury*_ = 34% of connections from the trained weight matrices (Figure 9A), by setting random connections to 0 in the weight matrix (calculated as average percentage of boutons lost via the injury). After injury, we update the weight matrix to simulate the results we observed in the live animal experiments (for example of weight matrix modification, see Figure 10). In the aged EPB model, *Bgain*_*i*1_ = 18.8% connections are gained (Connections are added with weights randomly initialised between 0 to w_init_, and size is increased by *Bsize*_*i*1_ = 1.22 (stage 1). This is followed by *Bloss*_*i*2_ = 18.8% connections are lost (connections are removed by setting weights to 0), and size is decreased by *Bsize*_*i*2_ = 1.22 back to baseline levels (stage 2). In addition, the new connections from the previous step are increased by *BsizeNew*_*i*2_ = 2.41, as seen in the experimental results in this paper (stage 2).

1. In the young EPB model, *Bgain*_*i*1_ = 2.5% connections are gained
2. In the young TB model, *Bgain*_*i*1_ = 26% connections are gained, and additional

*Bloss*_*i*2_ = 1% of connections are lost post-lesion.

After each step (update to the weight matrix), we conducted a single repeat of the training simulation (using the same parameters as during training but with one repeat instead of four, incorporating Hebbian learning) to mimic dynamic brain conditions and the passage of time. Subsequently, we performed an associative memory task (as previously described) to assess performance. Our goal was to determine whether recovery from injury is feasible under these synaptic dynamics.

##### Membrane potential dynamics

The leaky integrate and fire model^72^^,^^73^ that we used is defined below (Table 3):

(Equation 2) $$\tau_{m}\frac{d}{dt}u\left(t\right)=-\left[u_{\left(t\right)}-u_{rest}\right]+RI\left(t\right)$$

The internal current is updated at each time-step according to neuron firing:

(Equation 3) $$I\left(t\right)=\sum_{\left\{i\right\}}w_{\left\{ij\right\}}\;\exp\left(-\frac{\left(t-t^{f}\right)}{\tau_{s}}\right)$$

(Equation 4) $$t^{f}:u\left(t^{f}\right)=\theta \text{,}$$

where *u*(*t*) is the time-varying membrane voltage, *u*_*rest*_ is the resting membrane potential; *R* is the membrane resistance, *τ*_*m*_ is the membrane time constant, *τ*_*s*_ is the spike time constant, *I*(*t*) is the associated time-varying current, *w*_*ij*_ is the synaptic strength between presynaptic neuron *i* and postsynaptic neuron *j*, *t*^*f*^ is the *f*^*th*^ firing time, and *θ* is the firing threshold voltage. A reset condition is also applied if the membrane potential goes over a threshold, *θ* (*−*38 *mV*):

(Equation 5) $$u\left(t^{f}\right)=u_{rest}\text{.}$$

##### Plasticity

We implemented a rate based Hebbian learning rule.^28^^,^^74^ A weight change, *dw*_*ij*_*/dt*, is applied depending on the firing rates of presynaptic and postsynaptic neurons (*ν*_*i*_*, ν*_*j*_, respectively):

(Equation 6) $$\frac{dw_{\left\{ij\right\}}}{dt}=F\left(\eta ,v_{i},v_{j}\right)\text{,}$$

where *η* is the learning rate, and *F* is the learning function which is shown in the equation above and summarised in Table 4.

The firing rate of each neuron *ν* is updated at each time-step:

(Equation 7) $$\tau_{\nu }\frac{d}{dt}\nu \left(t\right)=-\nu \left(t\right)+\exp\left(-\frac{\left(t-t^{f}\right)}{\tau_{s}}\right)$$

The weight update follows a mechanism similar to the covariance rule,^74^ but incorporates a Heaviside function:

(Equation 8) $$\frac{d}{dt}w_{i,j}=\eta \left(v_{i}-\left\langle v_{i}\right\rangle \right)\left(v_{j}-\left\langle v_{j}\right\rangle \right)\left(1-H\left(-\left(v_{i}-\left\langle v_{i}\right\rangle \right)\right)H\left(-\left(v_{j}-\left\langle v_{j}\right\rangle \right)\right)\right)$$

(Equation 9) $$H\left(x\right)=\left\{ \begin{array}{cc} 0, & if\;x<0\\ 1, & if\;x\geq 0 \end{array}\right.$$

where *τ*_*ν*_ is the time constant of low-pass filtered spike train, *H* is the Heaviside step function, *(ν)* is the average threshold for the firing rate for a neuron over time window *t*_*w*_ (i.e., the firing rate threshold is updated as a running average over recent history), and *η* is the learning rate. The weight between two neurons, *w*_*ij*_ is bounded so that it is kept between a lower and an upper bound,

$$w_{\min}<w_{ij}<w_{\max}\text{.}$$

#### Parameter fitting

To select suitable parameters for the EPB and TB models, we referenced the connectivity and biophysical properties of the somatosensory cortex^29^ (Table 3). Since TB-rich axons originate from L6 and EPB-rich axons from layers 2/3/5 and the thalamus, we assigned biophysical properties accordingly from these respective layers. EPBs are reported to be larger and more stable,^30^ so we assigned them a higher weight boundary (reflecting their size) compared to TBs, with weight boundaries set to 1.28 for EPBs and 0.64 for TBs (Table 6). Another property we tried to keep within the physiological range is the firing rate, which has been reported to average 11 ± 0.82 Hz,^69^^,^^70^ with a maximum of 185 ± 10 Hz for neurons in the somatosensory cortex.^69^^,^^71^ For both the aged and young models, we fitted the TOR (using the same calculation as in the experiments) for young (EPBs and TBs) and aged (EPBs) synapses.^11^^,^^12^^,^^19^ TOR is computed based on gains and losses-i.e., a gain occurs when the weight goes above the *θ*_*synaptic*_ threshold, and a loss occurs when the weight goes below it (Table 5).

We computed TOR at each simulation for different learning rates (*β*). We then made sure that the TOR of each group (e.g., young adult EPB, and aged EPB) was in the correct ratio to each other, within an error margin of 0.01. For instance, in the aged EPB group, the TOR from experimental methods is 0.15, while in the young EPB group is 0.08. For each pair of learning rates, we verified that the TOR in the aged group was 1.85 times higher than in the young group. We performed the same process for the other group comparisons (aged EPB vs. young TB; young EPB vs. young TB). The model parameters used for the injury simulation are summarized in Table 2. We first extracted parameters from existing publications,^11^^,^^12^ where we computed that following injury, EPB-rich axons regenerate and gain boutons equal to the number of boutons lost, divided by 13.7. This was computed by looking at the ratio of number of boutons lost due to injury, to number of boutons gained during regeneration. In a similar manner, we also computed this ratio for TB-rich axons and found that the number of boutons gained following injury is equal to the number of boutons lost divided by 1.29, indicating that there is more regeneration in TB-rich axons. In TB-rich axons, there was also a pruning mechanism following injury, where 0.1 of the boutons were lost. We then conducted a comprehensive analysis of the bouton dynamics shown in this paper to incorporate them into the model. We split the simulation of the aged EPB axons into two stages (see Table 2 for a complete breakdown of each stage): (1) the first stage for the 6-h time-point, where we observed an increase in both the number and size of boutons, and (2) the second stage, where these values returned to baseline. More specifically, we found that 6 h following injury, there was a 1.22-fold increase in the size of boutons and a 1.188-fold increase in the number of boutons (calculated based on the boutons currently present). The newly gained boutons were observed to increase their size by a factor of 2.41 before injury. For readability in the results section, we report only the result from the second stage.

### Quantification and statistical analysis

We performed all statistical analyses using SPSS or MATLAB. Before conducting statistical tests, we assessed the normality of the data. Based on this, we selected appropriate statistical tests. A significance level of *p* < 0.05 was used for all tests, which were all two-tailed. A binomial test was used to assess whether the observed lack of regeneration differed significantly from the expected rate based on young neurons. For the rest of the multiple comparisons, we applied a Bonferroni correction, adjusting the significance level to 0.05/n, where n is the number of tests performed (only *p*-values under this threshold were considered significant). For all figures, the error bars represent the Standard Error of the Mean (SEM).

## Acknowledgments

Claudia Clopath for leading and supervising the computational modeling efforts. Emma Mustafa, Aleksandra Czerniak, Katie Horan, Aaron Matthews, and Emma Rowley assisted with animal care and monitoring. Sera Singha Roy for helpful discussions and for preparing the Graphical Abstract and STAR Methods. Funding: Supported by the 10.13039/501100000265Medical Research Council (V.D.P.) and the Engineering & Physical Sciences Research Council Doctoral Training Program in Neurotechnology (V.D.P, C.B., A.A.B.).

## Author contributions

C.B. collected all data, conducted the analysis, developed the computational model, and wrote the original draft. A.A.B. co-supervised the project and interpreted the data. V.D.P. led the supervision, conceptualized the project, developed the methodology, performed cranial window surgeries, interpreted data, and reviewed the article. All authors contributed to the article and approved the submitted version.

## Declaration of interests

The authors declare no competing interests.

## Supplemental information

Document S1. Figures A1 and A2

## Footnotes

Supplemental information can be found online at https://doi.org/10.1016/j.isci.2025.113665.
