# Supplementary material for: Impaired axon regeneration and heightened synaptic dynamics in the injured aged mammalian cortex
Source: iScience. 2025 Sep 30;28(11):113665. doi: 10.1016/j.isci.2025.113665 (PMC12613038; doi:10.1016/j.isci.2025.113665)
Supplement: Document S1. Figures A1 and A2 [file mmc1.pdf]

iScience, Volume *28*

Supplemental information

Impaired axon regeneration and heightened synaptic dynamics in the injured aged mammalian cortex

Cher Bass, Anil A. Bharath, and Vincenzo De Paola

Additional Injury Simulation Experiments

We performed an additional baseline experiment to explore whether re-wiring following injury occurs non-optimally. Specifically, in this baseline condition, boutons gained following injury were added randomly across the entire weight matrix, and similarly, boutons removed were eliminated randomly from the weight matrix (Fig. S1).

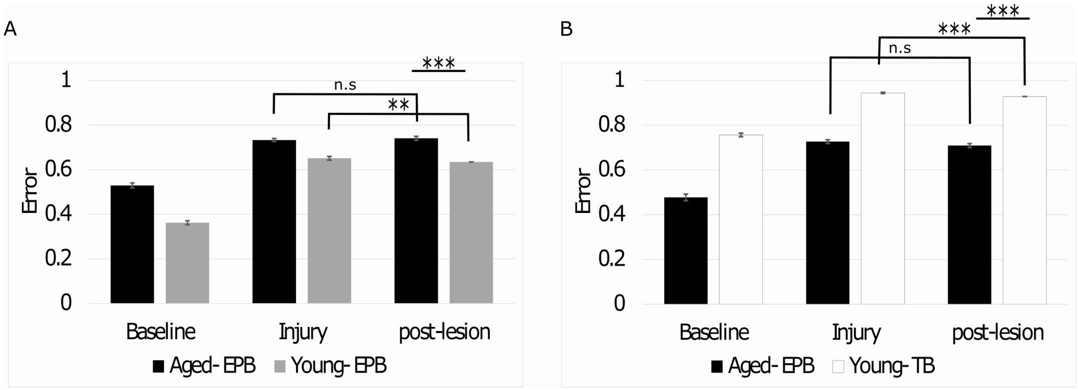

Figure S1. Results for injury simulation with random wiring.
A, No difference between injury and post-lesion in the aged EPB condition, recovery of 0.02 (p < 0.01) in the young EPB condition. **B**, No difference between injury and post-lesion in the aged EPB condition, recovery of 0.02 (p < 0.001) in the young TB condition. Error bar, SEM.

As expected, recovery was less pronounced in the non-optimal re-wiring condition. However, we still observed significant recovery in the young EPB (p < 0.01) and young TB (p < 0.001) conditions. In contrast, recovery was not significant in the aged EPB (p = 0.09) condition. These results suggest that the brain likely performs re-wiring in a more optimal manner when its aim is to recover synaptic dynamics. The true re-wiring mechanism may lie somewhere between the random and optimal re-wiring strategies we tested in these simulations.

Time Step Validation

We validated the correctness of our model by altering the time step (dt) by a small amount (dt = 0.1, 0.095). We compared the training and testing simulation graphs, weight matrices, and firing rates across five repeats and found them to be comparable. During training (Fig S2, left), the average firing rate was 7 ± 0.4 Hz and 9.5 ± 0.9 Hz for dt = 0.095 and 0.1, respectively. The firing rates during testing were also comparable (Fig S2, middle). Additionally, the sum of the weights, averaged over five repeats, was nearly identical: 0.1772 ± 0.01 for dt = 0.095 and 0.1778 ± 0.01 for dt = 0.1 (Fig S2, right). This demonstrates that our model correctly normalizes the simulations with respect to dt. A large change in dt is likely to affect the results, as the model's accuracy decreases with larger changes in dt. In contrast, smaller changes in dt lead to higher accuracy.

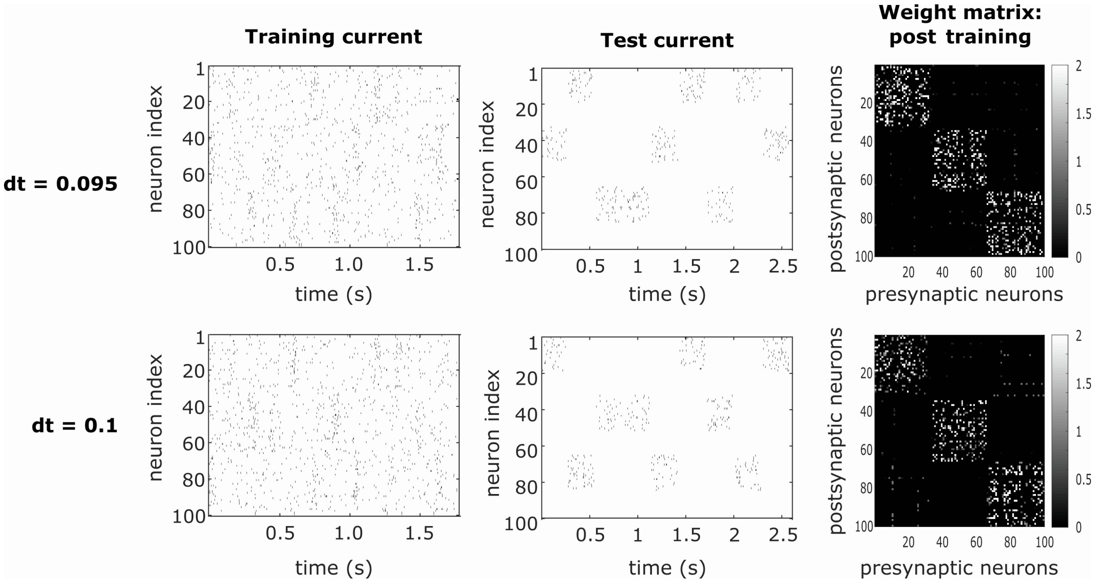

Figure S2. Plots for simulations with varying *dt*s.
Example training and testing current plots, and weight matrices for each simulation, with different *dt*s (0.095 and 0.1).
